# Emerging role of extracellular vesicles in the respiratory system

**DOI:** 10.1038/s12276-020-0450-9

**Published:** 2020-06-15

**Authors:** Joshua Holtzman, Heedoo Lee

**Affiliations:** 1 Department of Biology, Oberlin College, Oberlin, OH USA; 2 Department of Biology and Chemistry, Changwon National University, Changwon, Korea

**Keywords:** Respiratory tract diseases, Respiration

## Abstract

Extracellular vesicles (EVs) present numerous biomedical ways of studying disease and pathology. They function as protective packaging for the delivery of controlled concentrations of miRNAs and effector molecules, including cytokines, chemokines, genetic material, and small signaling molecules. Previous studies of EVs have yielded valuable insights into pathways of intercellular communication that affect a variety of biological processes and disease responses. The roles of EVs, specifically microRNA-containing EVs (EV-miRNAs), in either mitigating or exacerbating pulmonary disease symptoms are numerous and show promise in helping us understand pulmonary disease pathology. Because of their well-documented involvement in pulmonary diseases, EVs show promise both as possible diagnostic biomarkers and as therapeutic agents. This review surveys the physiological functions of EVs in the respiratory system and outlines the pulmonary disease states in which EVs are involved in intercellular crosstalk. This review also discusses the potential clinical applications of EV-miRNAs in pulmonary diseases.

## Respiratory system: extracellular vesicles in health and disease

Studies of tiny membrane-bound sacs called extracellular vesicles (EVs), which bud from cells naturally but are also implicated in disease, offer insights into respiratory health and disease, and could be used to deliver therapies into respiratory system cells. Joshua Holtzman at Oberlin College, Ohio, USA, and Heedoo Lee at Changwon National University in South Korea review current understanding of the role of EVs in the respiratory system and their potential uses in treatment. Researchers are discovering how EVs deliver signaling molecules to promote respiratory health, and how they can be involved in cancer, autoimmunity, asthma and other diseases. Early trials using EVs to deliver conventional drugs, and small RNA molecules that can control gene activity suggest great potential for treating a range of serious respiratory conditions. Analysis of EVs may also assist in diagnosis.

## Introduction

### What is an extracellular vesicle?

Extracellular vesicles (EVs), also known as exosomes or microvesicles, are a family of exocytotic particles composed of phospholipid bilayers that carry a variety of nucleic acids, lipids, cytosolic proteins, receptors, and other small signaling molecules^1^. Ranging from less than 50 nm to 1000 nm, these membrane-derived vesicles have functions from intracellular signaling to quorum sensing, and recent evidence suggests their critical involvement in basic cell functions such as inflammatory defenses, sharing of genetic and epigenetic information, and more^2,3^. However, studies also suggest that they are involved in a variety of cancers, autoimmune diseases, and cellular dysfunction^2,4^. Most EVs are derived from the phospholipid bilayers of cells, as part of either endocytic or programmed cell death pathways. EVs show promise not only as therapeutic and drug-delivering treatments but also as markers of disease and inflammation in pulmonary dysfunction due to their involvement in the pathogenesis of many pulmonary diseases.

### Three types of EVs

EVs are highly heterogeneous and vary in size, morphology, and content. However, EVs fall into three distinct classes: apoptotic bodies, microvesicles, and exosomes. Each type of EV has its own fundamental functions and characteristics. For example, apoptotic bodies (ABs) are produced when programmed cell death occurs and can range from 50 nm in size to upwards of 5,000 nm^5^. Their formation via the blebbing of cytosolic content from dying cells means that these vesicles vary greatly not only in size but also in content. Their surface markers, which include histones, TSP, C3P and exposed phosphatidylserine, among others, can be recognized by most cell types, specifically phagocytic cells^5^.

**Fig. 1:**
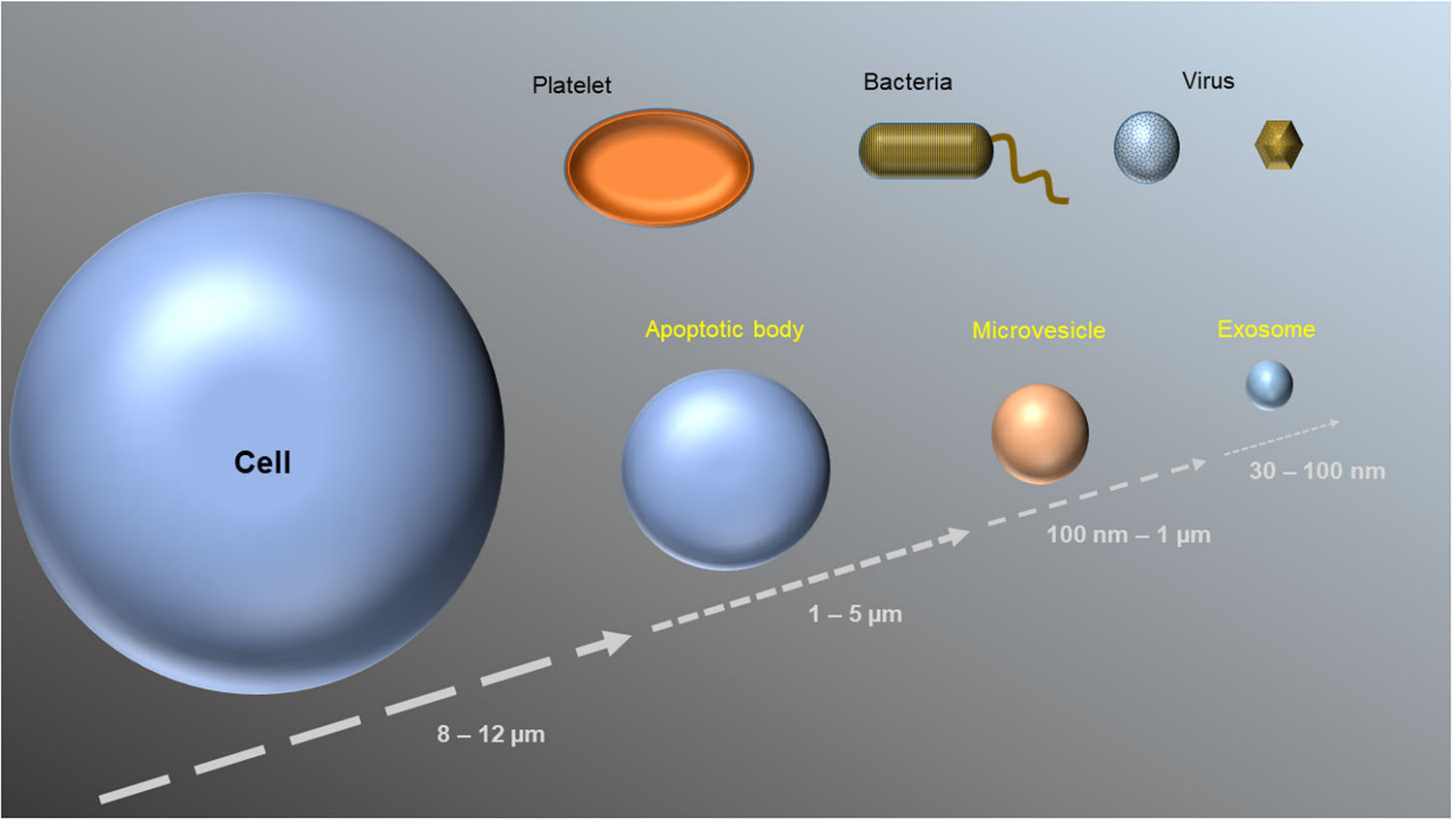
Size distributions of EVs derived from mammalian cells. The sizes of three types of EVs are compared to those of other small structures, including cells, platelets, bacteria, and viruses.

**Fig. 2:**
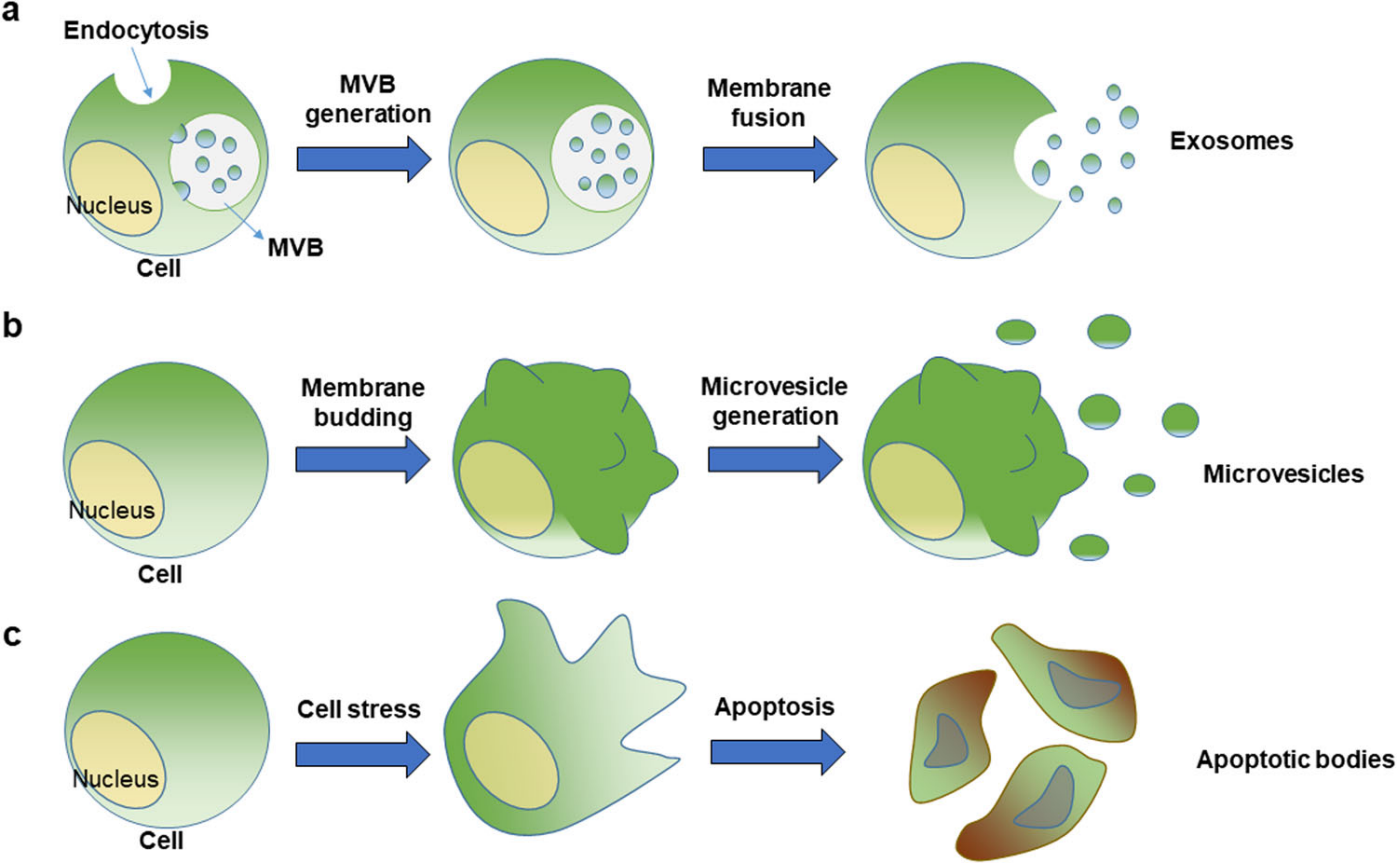
Generation mechanisms of three types of EVs. **a**–**c** The generation processes of exosomes, **b** microvesicles, and **c** apoptotic bodies are mechanistically different in mammalian cells.

Microvesicles (MVs) and exosomes, on the other hand, belong to a category of EVs that carry deliberate cargo to their targets. MVs are formed via direct budding of the plasma membrane^1^. They are typically between 200 nm and 1000 nm in size and are often miRNA-rich^6^. Recent studies have shown that RNA-binding proteins, such as hnRNPA2B1 and the lipid raft protein caveolin-1 regulate the sorting of miRNAs into MVs^7,8^. Exosomes are the smallest EVs, usually less than 100 nm^1,2^. Exosomes are different because they are prepackaged in multivesicular bodies (MVBs) and exocytosed once the MVE fuses directly with the plasma membrane, instead of budding from the plasma membrane like MVs^1^. Exosomes and MVs share many surface markers, such as flotillin, caveolin, and several tetraspanin/CD protein markers. This makes it hard to differentiate them biochemically, as well as with microscopy and flow cytometry^6^. However, subtle differences between marker concentrations and composition between MVs and exosomes can be found in cells with different physiological functions^9^. The sizes and generation mechanisms of the three types of EVs are graphically summarized in Figs. 1 and 2.

### Suggestions of the ISEV on the nomenclature of EVs

The International Society for Extracellular Vesicles (ISEV) has outlined extensive suggestions for the nomenclature of EVs called the Minimal Information for Studies on Extracellular Vesicles (MISEV)^10^. The MISEV suggests rigorous use of operational terms in naming newly discovered EVs, specifically terms that describe the physical characteristics, biochemical composition, conditions, or cell origin of the EV in question^10^. For example, CD9-positive EVs, lung epithelial cell-derived EVs, and oxidative stress-induced EVs can be named as specific populations of EVs.

In addition, all experimental conditions through which the EVs and their characteristics were identified should be diligently documented in the “Methods” section of the primary report. These descriptions should contain information regarding the cell culture conditions, EV isolation techniques, and instrumental/experimental settings for characterizing EVs^10^.

### General functions of EVs

Because of their pervasiveness in most cell types and ability to mobilize a wide range of cargo to a variety of targets, the various functions of EVs are widespread and are just beginning to be understood. EVs are known to be an alternative to the classical “constitutive” exocytic pathway, in which the cargo is excreted from the cell through intercellular vesicles that fuse to the plasma membrane of the cell and directly release the cargo outside the cell. Advantages to nonconstitutive “granule-mediated” exocytic packaging and release are apparent, enabling protection of the cargo, guided targeting towards its destination, and expedited control over the plurality and strength of the signal or vesicle content that is sent^3^. Although cytokines mostly take advantage of constitutive exocytosis, EVs have been shown to carry, mediate, and regulate cytokine transport from cells to members of the immune system.

In addition, EVs can carry controlled profiles of miRNAs^11–13^, which can directly influence gene expression in recipient cells. miRNAs are a class of evolutionarily conserved short noncoding 19- to 22-nucleotide regulatory RNAs^14,15^. miRNAs have been found to impact gene expression posttranscriptionally. The biological functions of EV-associated miRNAs have been extensively studied, and EV-associated miRNAs are now recognized as an essential factor for intercellular communications in various disease conditions^11,14,16–19^. We will discuss this topic in the latter part of this paper.

## Extracellular vesicles in the respiratory system

### History of EV research in the respiratory system

Although vesicles are a well-known aspect of cellular biology, EVs are a relatively recent addition to modern cellular and molecular biology. Extracellular vesicles and microparticles of the same nature were originally described by Chargaff and West in their study of procoagulant platelets, in which they observed secreted microparticles on the nanometer scale that aided in platelet procoagulant properties^3,20^. Since then, EVs have been better defined, found in most mammalian cell types, and linked to various diseases and physiological functions. Historically, the involvement of EVs in pulmonary diseases has been studied via the extraction of EVs from bronchial-alveolar lavage fluid (BALF) and lung-derived cells^4–10,21–23^. It is now well known that pulmonary EVs are sourced from various cell types, including lung epithelial cells, macrophages, and pulmonary endothelial cells^4–10,21–23^.

Because of studies in acute lung injury/inflammation, EVs, specifically MVs, are thought to help recruit M1 macrophages to damaged epithelial cells, demonstrating their importance in initiating and maintaining inflammatory responses in the pulmonary epithelium^9,21,24^. In 2007, EVs containing miRNAs (EV-miRNAs) and other noncoding RNAs were first discovered^14^. Interestingly, EV-miRNAs are suspected to be released during sterile and nonsterile lung damage in mice^6,8,9,25^ and are significantly involved in the development of lung inflammation^6,25^. Studies in hyperoxia-associated oxidative stress have demonstrated a dramatic shift in EV-miRNA profiles in vivo after hyperoxia is induced^7^. Specifically, more miRNAs are produced, and more MVs than exosomes carry them in the respiratory system^6,8,9^. In addition, Chevillet et al. reported that less than one miRNA is found on average in a single exosome^26^.

### Prominent sources of EVs in the respiratory system

EVs are especially instrumental in both normative organismal function and disease pathogenesis in the respiratory system. Recent studies have shown that EVs are primarily generated by lung epithelial cells during lung inflammation^9,21,24,27^. These lung epithelial EVs, composed mainly of miRNA-containing MVs, carry inflammatory signals that primarily target alveolar macrophages (AMs). These AM-targeting MVs^21,24^, sometimes EV-miRNAs^6,9^, upregulate cytokine receptors involved in NF-kB-mediated inflammation in recipient macrophages, such as TLR2 and TLR6. It is thought that this MV-mediated signaling classically activates macrophages to the M1 phenotype. These discoveries were facilitated by studying BALF-derived EVs in various lung disease conditions and analyzing the lung epithelial cells that secrete EVs and EV-miRNAs.

EVs can also be produced by hematopoietic cells such as monocytes, macrophages, and neutrophils in the respiratory system. The secretions of hematopoietic cell-derived EVs are strongly triggered by bacterial lung infection^21,25^ and allergic lung inflammation^27^. Notably, they secrete EV-miRNAs containing different profiles of miRNAs that significantly alter the local tissue environment and activate the innate immune responses by transferring functional miRNAs to the target recipient cells^21,25,27^. Moreover, a recent report has shown that polymorphonuclear leukocyte-derived EVs are coated with neutrophil elastase^28^. This EV-associated neutrophil elastase significantly degrades the extracellular matrix and causes alveolar destruction in the respiratory system^28^.

Moreover, endothelial cell-derived EVs are actively generated from the pulmonary endothelium. Functions of pulmonary endothelial cells are essential to maintain lung homeostasis^28^ and are frequently disrupted in various lung diseases, including pulmonary hypertension and acute/chronic respiratory failures^29–31^. Notably, it has been reported that damaged endothelial cells actively release EVs and that endothelial EVs are known to regulate lung inflammation and the proliferation and apoptosis of pulmonary artery smooth muscle cells^29,30^. However, the specific roles of these endothelial EVs in lung diseases remain to be clearly elucidated.

## Physiological functions of EVs in respiratory diseases

### EVs in acute lung injury and acute respiratory distress syndrome

EVs have been found to have roles in many respiratory diseases. EVs have been most extensively studied in acute lung injury (ALI) and acute respiratory distress syndrome (ARDS)^32,33^. Previously, in studies of both sterile and nonsterile/LPS-induced lung injury, such as the aforementioned diseases, damaged or threatened epithelial cells were shown to communicate and recruit AMs to clean up the crime scene and engulf any pathogens that may have caused the damage as well as residual apoptotic bodies that would otherwise wreak havoc on the surrounding tissue^34,35^. Unfortunately, in ALI and ARDS, this inflammatory response is overwhelming and is the cause of symptoms^36^. Until recently, the pathways by which epithelial cells communicate with AMs and how AMs interface with each other were only understood at a base level. A recent study by Lee et al. presented functional evidence that EVs mediate this cellular crosstalk not only between epithelial cells and AMs but also between AMs and other AMs^21,25^. In both hyperoxia-induced and nonsterile-induced ALI/ARDS in mice, an increase of MVs filled with controlled concentrations of inflammatory molecules was found to be sent to nearby AMs after provocation^21^. Both sterile and nonsterile methods of inducing acute lung damage in mice showed different MV profiles, and each mode of inducing lung injury upregulated different processes inside the recipient AMs. For sterile stimuli-induced BALF EVs, effectors such as TLR2, IL-6, TNF-a, and Myd88 were significantly upregulated. In contrast, infectious stimuli-induced BALF EVs were responsible for the induction of TLR-6, IL-1b, IL-10, and CD80 expression in recipient AMs^21^.

### EVs in chronic obstructive pulmonary disease

The role of EVs in chronic obstructive pulmonary disease (COPD) is also well supported. COPD is another pulmonary disease spurred by chronic inflammation in the lung. This causes exacerbations such as significant airflow disruption, overproduction of mucus, and defective mucociliary clearance in the airways as a result of bacterial respiratory infections^37^. EVs are believed to be involved in the pathogenesis and exacerbation of COPD. In a study by Takahashi et al., a dramatic increase in endothelial cell-derived MVs was observed in stable COPD patients, suggesting physiological functions of MVs in vivo, such as intercellular communications^38,39^. In another study, increased exosome levels were also observed, and evidence suggested that these exosomes were involved in the inflammatory processes of COPD exacerbations^40^. EVs were also found to mediate leukotriene conversion in the affected tissue and regulate the pathogenesis of COPD^5^.

COPD is also characterized by airway remodeling and skeletal muscle dysfunction and is often caused by smoking-induced airway inflammation^41^. An increased concentration of miR-210 was found in the bronchial epithelial cell-derived EVs of patients with COPD^42^. These EV-miR-210 directly controlled the autophagy functions and differentiation of myofibroblasts^42^. Autophagy dysregulation promotes the production of reactive oxygen species, which are involved in COPD progression^43,44^. It was also reported that the number of EV-miR-21 was significantly upregulated in COPD patients, suggesting the potential application of EV-miR-21 as a diagnostic and therapeutic biomarker for COPD^45^.

### EVs in pulmonary hypertension

Pulmonary arterial hypertension (PAH) and its symptoms are characterized by the dysfunction of pulmonary arterial endothelial cells (PAECs), which leads to deadly symptoms such as right ventricular overload and right heart failure^46^. It is believed that EVs are critical in the communication between PAECs that initiates PAEC proliferation, eventually leading to the pathogenesis of PAH. In a study by Zhang et al., hypoxia, a precursor of PH, increased the secretion of 15-LO2-enriched exosomes^47^. 15-LO2 was found to cause in vitro proliferation of PAECs by activating their STAT3 pathway^47^.

A recent study also showed that miR-143-enriched exosomes are produced by pulmonary arterial smooth muscle cells (PASMCs) in the pathology of PAH. These exosomes are known to induce the migration and angiogenesis of PAECs, suggesting intercellular communication between PAECs and PASMCs via transfer of EV-miR-143 in the pathogenesis of pulmonary hypertension^48^.

### EVs in lung fibrosis

Idiopathic lung fibrosis (ILF) is a lethal interstitial lung disease that is caused by inflammation-exacerbated damage to lung epithelial cells^49^. Inappropriate myofibroblast activation causes brunt damage to lung epithelial cells in ILF^49^. Similar to some of the lung diseases mentioned above, an increase in EVs secreted by lung epithelial cells has been correlated with ILF. According to a study by Martin-Medina et al., EVs extracted from patients with ILF primarily contain the signaling protein WNT5A. WNT5A and the associated signaling pathway are known to contribute to disruption of lung epithelial cell homeostasis during ILF. Lung fibroblasts were identified as a major source of EV-bound WNT5A, and these EVs were found to promoted lung fibroblast proliferation and thus the pathology of the disease^50^.

In addition, exosomes released from mesenchymal stem cells have similar functions as their mother cells, which are involved in repairing damaged tissues and suppressing inflammatory processes^51^. It has been reported that exosomes are cytoprotective in pulmonary fibrosis^52,53^. However, the functional mechanisms of mesenchymal stem cell-derived exosomes are not fully understood and remain controversial^54^.

### EVs in asthma

Asthma features chronic recurring inflammation and subsequent obstruction of the airways as a result of bronchial hyperresponsiveness and sensitivity to inflammatory and allergenic particles^55^. Currently, it is considered chronic and incurable. Copious research has linked an array of miRNAs, likely released in EVs or other carrier molecules, with asthma. EV-miR-145 has been identified as essential to epithelial and smooth muscle cell functions during asthma-induced inflammation^56,57^. During asthma, miR-145 is inhibited, preventing eosinophilic inflammation, mucous hypersecretion, Th2 cytokine production, and airway hyperresponsiveness, all of which are physiological responses to bronchial stress such as that induced by asthma^41,57^. However, over 140 other plasma and sputum EV-miRNAs have been found to promote asthma symptoms and asthma-associated damage. For example, the levels of EV-associated miR-629-3p, miR-223-3p, and miR-12-3p are increased in the sputum of severe asthmatic patients^58^. In addition, plasma-derived miR-155 can be found in relatively large concentrations in asthma-induced inflammatory responses and ovalbumin (OVA)-induced airway inflammation and has been identified as a target for the transcription factor PU.1, which negatively regulates type 2 cytokine production and is targeted by Th2 immune cells^59–61^. miR-155 has also been found to impair ILC proliferation and the release of IL-33^62^. Other potential plasma-EV biomarkers of asthma include miR-125b, miR-16, miR-299-5p, miR-126, miR-206, and miR-133b^63,64^.

EVs containing signaling molecules in addition to these miRNAs can be derived from a host of immune cells and respiratory tissue. For example, exosomes from mast cells, T cells, dendritic cells, and eosinophils are associated with promoting the asthma-induced lung inflammatory response^65,66^. In addition, EVs that are sourced from fibroblasts can promote smooth muscle tissue and epithelial cell proliferation in severe asthma^67^. In contrast, some EVs, such as those sourced from mesenchymal stem cells, can upregulate immunosuppression and promote airway remodeling during asthmatic attacks^68^. Some lineages, such as mast cells, B cells, and mesenchymal stem cells, release EVs that recruit or regulate the proliferation of other cells, such as T cells^66^. The physiological functions of EVs in respiratory diseases are summarized in Table 1.

**Table 1:** List of respiratory diseases associated with EV functions.

Disease	EV Involved	Reference
ALI/ARDS	Lung epithelial cells produce MVs and mediate lung inflammation	^9,24^
	EVs facilitate the intercellular crosstalk between lung epithelial cells and alveolar macrophage.	^21^
COPD	Endothelial cell-derived EVs are upregulated in the lung.	^38,39^
	EV-miR-210 controls autophagy functions and differentiation of my fibroblast	^42^
Pulmonary hypertension	15-LO2-enriched exosomes trigger proliferation of PAECs by activating STAT3 pathway	^47^
	miR-143-enriched exosomes induce migration and angiogenesis of PAECs,	^48^
Lung fibrosis	EV-bound WNT5A promote lung fibroblast proliferation and pathogenesis of lung fibrosis	^50^
	EVs released from mesenchymal stem cells are involved in repairing damaged tissues	^51^
Asthma	EV-miR-145 is essential to epithelial and smooth muscle cell functions during asthma-induced lung inflammation	^56^
	EVs from mast cells, T-cells, dendritic cells, and eosinophils promote asthma-induced lung inflammation	^65,66^
	EVs from fibroblasts promote smooth muscle tissue and epithelial cell proliferation in severe asthma	^67^

## Clinical applications of pulmonary EVs

### Pulmonary EV-miRNAs

EV-associated miRNAs are ways by which cells can directly influence gene expression in recipient cells^14^. EV-miRNAs show promise as a tool in diagnosing diseases and their pathology^69^. miRNAs are short noncoding regulatory nucleotides that can regulate gene expression posttranscriptionally and modulate diverse biological processes, such as cell differentiation, proliferation, migration, survival and death^70^. Since they are relatively stable in serum and are often overexpressed in many diseases, including cancer and acute lung injuries, they have been recognized as excellent biomarkers for diseases^69,71^. Notably, different profiles of EV-miRNAs have been linked to diverse disease states^7,8,70,72–75^, and many reports have shown that RNA-binding proteins, such as Argonaute proteins and heterogeneous nuclear ribonucleoproteins (hnRNPs), are involved in the sorting of selected miRNAs into EVs^12,13,76–80^.

In studies of pulmonary EV-miRNAs, Lee et al. demonstrated that hnRNPA2B1, which has been shown to control the maturation of miRNAs, binds selective miRNAs in response to oxidative stress and guides these miRNAs into EVs for secretion^8^. Interestingly, they also found that caveolin-1, a lipid raft protein, is exclusively expressed in pulmonary EVs in which miRNAs are highly concentrated^6^ and efficiently escorts the hnRNPA2B1-miRNA complex to EVs^7,8^. Moreover, posttranslational modifications, such as Y14 phosphorylation of caveolin-1 and O-GlcNAcylation of hnRNPA2B1, are essential for the sorting of specific miRNAs into EVs^7,8^. These results suggest that the transfer of miRNAs between cells and EVs is tightly regulated in the respiratory system.

### Pulmonary EV-miRNAs as a new type of biomarker

EV-miRNAs, because of their ability to reflect host cell conditions and motive, have been isolated as a potential new type of biomarker^69^. In addition, because of the stability provided by the vesicles, EVs have become a more reliable source of marker miRNAs^69,71^. EVs also mediate the specific delivery of miRNAs through targeted fusion with the recipient cell’s plasma membrane^81^. Recently, a subpopulation of EV-miRNAs found in BALF that are rich in the previously discussed lipid raft protein caveolin-1 have been found to affect the phenotype of recipient AMs to promote inflammation, making these EV-miRNAs ideal markers for pulmonary diseases that are characterized by excessive inflammation^6^. Although EVs vary in density, they can be separated by density-dependent fractioning, making for ease of use as a general biomarker. Combined with detecting the presence of higher-density proteins such as caveolin-1 and flotillin, which indicates that miRNA loading has taken place in lung epithelial cells, identifying EV-miRNAs based on miRNA composition is becoming a novel way to diagnose pulmonary diseases via density and chemical markers^6^.

However, there are issues with using EV-miRNAs as biomarkers. Despite emerging evidence of biochemical differences between exosomes and microvesicles, their sizes and biological attributes tend to overlap, and current EV isolation methods may not be sufficiently discriminatory to separate the two subtypes of EVs. The main problem herein is that it is estimated that less than one copy of miRNA may be found per exosome^26^, but far more miRNA may exist in microvesicles^6^, making them ideal markers for miRNA composition.

### Pulmonary EV-miRNAs as a new type of therapeutic agent

Naturally, EVs have been considered a novel way of transporting drugs^82,83^. Since these vesicles protect, stabilize, and properly transport their cargo to recipient cells, cell-derived and synthesized EVs show promise for the next generation of drug delivery methods^14,69,71^. Since they are made of similar material as the cell surface of the target cell and may contain surface proteins that facilitate entry into target cells, they are exceptionally good at being absorbed into host tissue^14,83^. EV-miRNAs facilitate miRNA delivery to target cells^14^. They can be either synthesized or, more commonly, serum-derived or host cell-derived^6,84^. Derivation of EVs from serum or host cells is more efficient because the EVs can be generated on a greater scale with a higher chance of uptake by target cells due to preexisting surface proteins and markers. EV-mediated miRNA delivery has another unexpected upside—their method of delivery varies with their size. Particles are normally deposited in the lung via one of three delivery methods: impaction, sedimentation, and diffusion. Particles less than 100–500 nm have to be deposited in the bronchioles via diffusion, aided only by Brownian motion^85^. Particles larger than 5 μm are deposited by impaction. Nanoparticles and liposomes that lie between 1 and 5 μm are deposited via sedimentation^86^. This makes it easier for exosomes than other carriers to be deposited further into the lungs in the alveolar region as opposed to the bronchioles^87^.

Novel methods are being developed that allow delivery of small RNAs, drugs, and inflammatory mediators via EVs. Exosome-mediated miRNA delivery is currently being explored after the success seen with exosome-mediated siRNA delivery for Alzheimer’s disease treatment^88^. Zhang et al. reported a novel way to efficiently and selectively load miRNAs^84^. This enrichment utilized a modified calcium chloride transfection method in tandem with electroporation. The results were promising and presented a much more accessible and feasible way of controlling the transfection of miRNAs into exosomes^84^.

Exosome-mediated delivery of miRNAs is the most studied method of miRNA delivery. Exosomes show promise as controlled intratracheal purveyors of miRNAs^24,25^. Since they are endogenously generated, they are less toxic and immunogenic than other forms of nanoparticle-mediated drug delivery^87^. Since they are made of endogenous material, they can bypass the blood-brain barrier and other difficult biological barriers^89^. However, one of the issues with intratracheal exosome-mediated miRNA delivery into the lungs is that only AMs take up inhaled exosomes, not other phagocytes or even lung epithelial cells^87^.

miRNAs have been shown to be promising therapeutic agents; as discussed earlier in this review, miRNAs can affect target cells and, in the case of pulmonary diseases, can recruit and classically activate AMs^6,8,9,21,24^. Often having multiple target genes, miRNAs are relatively stable molecules compared to other RNAs but are still vulnerable to RNase digestion, and delivering them in effective concentrations can be difficult^84,87^. EV-miRNAs are a relatively novel method of transporting miRNAs to target cells, providing protection from the above threats and maintaining controlled concentrations of miRNAs directed to the target. Previously, the primary nanoparticles of choice for drug delivery were synthetic liposomes, which were used to deliver radiosensitive dyes more effectively. However, because they are synthetic, they have a low uptake in target cells^83^. Since then, serum-derived EVs have proven more stable and less offensive to host systems than synthetic liposomes^87^. As discussed above, novel methods of loading miRNAs of interest into exosomes and microvesicles are being developed, making EV-mediated miRNA and drug delivery more accessible than ever.

The study by Zhang et al. that perfected the enrichment of exosomes with miRNAs utilized miR-15a, an miRNA that is essential in regulating innate immunity and host defense, to test their loading strategies^84,87^. This successful technique indicates that EV-miRNAs are closer than ever to being used intratracheally as potential therapeutic drug delivery systems to treat pulmonary diseases. Other pulmonary miRNAs that mediate the respiratory diseases mentioned above could feasibly be loaded into EVs and delivered intratracheally to mediate disease progression. The biological functions of EV-miRNAs in the respiratory system are summarized in Table 2.

**Table 2:** List of EV-miRNAs in respiratory systems.

miRNA	Function	Reference
miR-17/93	Promote macrophage migration and secretion of TNF and IL-1β, possibly via downregulation of Irf2bp2	^8^
miR-223/142	Suppress Nlrp3 inflammasome activation in macrophages via inhibition of Nlrp3 or Asc	^25^
miR-17/221	Promote macrophage infiltration into the lung via induction of integrin β1 recycling	^24^
miR-221/222	Promote lung epithelial cell growth by modulating cyclin-dependent kinase inhibitor 1B (CDKN1B) pathways	^90^
miR-320a/221	promote macrophage-regulated lung inflammatory responses via NF-κB signaling pathway	^9^
miR-15a	Suppress macrophage-mediated lung inflammation, possibly via TLR-4 signaling pathway	^84,87^
miR-155	Regulate macrophage polarization via Akt-1 signaling pathway	^87,91^
miR-210	promote myofibroblast differentiation via modulating autophagy function (targeting ATG7)	^42^
miR-143	Induce pulmonary endothelial cell migration and angiogenesis via unknown mechanism	^48^
miR-145	inhibited eosinophilic inflammation, mucus hypersecretion, T(H)2 cytokine production, and airway hyperresponsiveness	^41,57^

## Conclusion

EVs are now considered a critical biological factor in the respiratory system. In addition to being a controlled mechanism of effector delivery, they have proven to be integral in both the pathogenesis and amelioration of lung diseases. Trials in intratracheally delivered EVs not only suggest their importance in these diseases but also show immense promise as a breakthrough medical tool to deliver medicine and effector miRNAs. EVs have opened up a whole new world of possibilities in pulmonary medicine and have opened the door to further exploration of pulmonary diseases and their mechanisms. However, there is still much more work to be done to understand and utilize EVs for the betterment of medicine. For example, the mechanisms by which mesenchymal stem cells and lung epithelial cells release EVs still need better elucidation, and better characterization of EV-miRNAs and their targets is needed, especially to identify them as fully dependable biomarkers. Further lines of research will clarify the way for improved medicine and diagnostics in pulmonary diseases.
